# Is chemodenervation with incobotulinumtoxinA an alternative to invasive chronic anal fissure treatments?

**DOI:** 10.1186/s12876-024-03428-z

**Published:** 2024-09-30

**Authors:** T. Calderón, L. Arriero, P. Cruz, L. Gómez, J. Asanza, J. C. Santiago, R. Garrido, C. Bustamante, T. Balsa

**Affiliations:** 1 Servicio de Cirugía General y Aparato Digestivo, Hospital Nuestra Señora del Prado. Talavera de La Reina, Toledo, Spain; 2 Servicio de Cirugía General y Aparato Digestivo, Hospital Universitario de Guadalajara, Guadalajara, Castilla-La Mancha Spain

**Keywords:** Botulinum toxin, Efficacy, Safety, Chronic anal fissure

## Abstract

**Background:**

Botulinum toxin type A is currently strongly recommended for the treatment of anal fissures (AFs). However, there is still no consensus on dosage or injection technique. This study provides further efficacy and safety evidence in a 2-year follow-up.

**Method:**

Prospective, open-label, single-arm, single-center study carried out in adult patients with AFs non-responsive to previous treatments. Patients were treated with incobotulinumtoxinA (incoBoNT/A) injected in both laterals and posterior intersphincteric groove. Healing rate at 2 years was the primary endpoint. Secondary endpoints included internal anal sphincter pressures, incontinence, and safety.

**Results:**

A total of 49 patients were treated with a mean incoBoNT/A dose of 40.5 U (spread across three locations). Healing rate at 2 years was 83.9% with a 24.5% of recurrence throughout the study. Only 7 patients (14.3%) reported adverse events (AEs) that were mild and temporary. Mean reduction in anal resting pressure was -9.1 mmHg at 3 months (*p* = 0.001). Mean reduction in voluntary squeeze pressure was -27.5 mmHg at 3 months (*p* < 0.001). Mean pain perception measured with a visual analog scale decreased by -6.5 points at 2 years (*p* < 0.001). There was an incontinence increase at 1 month of 1.3 points (*p* = 0.006), but baseline values were restored at 6 months.

**Conclusion:**

We present results that support the use of incoBoNT/A as a second line for AFs that do not respond to ointment therapy. IncoBoNT/A injection is a less invasive treatment that should be considered before surgery due to its efficacy and its safety which includes no permanent impairment.

**Trial registration:**

ISRCTN90354265; Registered on 16th February 2024. Retrospectively registered.

**Supplementary Information:**

The online version contains supplementary material available at 10.1186/s12876-024-03428-z.

## Introduction

Anal fissure (AF) is a tear in the skin of the anal canal that extends from the dentate line to the anal verge [1]. It is the most common cause of anorectal pain on defecation [2]. AFs are most commonly located in the posterior midline (73%) but can be found in the anterior midline in 13% of women and 8% of men, with 2.6% occurring both anteriorly and posteriorly simultaneously [3]. The overall annual incidence of AF is 0.11% [4] without sex differences, and it affects mainly young and middle-aged patients [5]. The main symptom associated with AF is anal pain (present in 90,8% of patients [6]) occurring during defecation and for several hours afterwards [5], that sometimes radiates to the buttocks, lower back, or upper posterior tights [7]. Moreover, bleeding appears in 71.4% of patients, and anal pruritus can also emerge [6]. The AF pathophysiology is not entirely clear, but trauma to the anal canal seems to be an important factor: constipation, diarrhea, vaginal delivery trauma, history of anal trauma or IAS hypertonia [5]. Patients with chronic AF exhibit higher resting pressures of the IAS than normal controls. Relative ischemia of the posterior commissure of the anal canal is another possible explanation for AF. Lower blood flow was found in the posterior midline than in the rest of the anal canal, which may also account for the predominance of fissures in the posterior midline [5, 7, 8]. There are also secondary AFs, caused by surgical anal procedures, infectious diseases, inflammatory diseases (Crohn’s disease), cancer, or sexually transmitted diseases [5]. These secondary AFs are considered atypical due to their location usually off the midline position. Several therapeutic options are available for AFs. The conservative option is considered as the initial approach and it is based on the hypothesis of constipation as the one of the causes of AF [3, 9]. Sitz baths and fiber supplementation are the core of this treatment, and topical steroids or anesthetics can be added. These dietary and behavioral modifications are considered safe since they entail few adverse effects, and they might heal the AF and even prevent recurrence if maintained [3]. When AFs persist, there are surgical (i.e., open and closed lateral internal sphincterotomy conventional or tailored, and anocutaneous advancement flap) and nonsurgical (pharmacological) options. The latter is based on achieving IAS transitional relaxation. It helps overcome hypertonia and favors vascularization of anal mucosa, allowing normal tone to be reached subsequently, and therefore avoiding incontinence [9]. The American Society of Colon and Rectal Surgeons (ASCRS) strongly recommends topical nitrates (ASCRS grade 1B: strong recommendation, moderate-quality evidence) [3], associated with healing of approximately 50% of chronic AFs [10]. With a superior side effect profile than nitrates, calcium channel blockers (CCB) have a similar efficacy and are also recommended as first-line treatment (ASCRS grade 1B) [3], with healing rates ranging 65–95% [11]. Botulinum toxin type A (BT) local injection is considered when topical therapy fails (ASCRS grade 1B: strong recommendation, moderate-quality evidence) [3, 9]. BT inhibits acetylcholine release at the neuromuscular junction, preventing neural transmission, and reducing anal sphincter tone at rest [12]. Moreover, BT reduces pain by inhibiting the release of other non-cholinergic neurotransmitters [13]. Local chemical denervation produced by BT begins 3 to 4 days after injection and fades gradually during the third or fourth month [13, 14]. The maximal benefit is detected 4–6 weeks post-injection [14]. BT is comparable to topical treatments as a first-line therapy for chronic anal fissures and shows modest improvement in healing rates when used as a second-line therapy after unsuccessful topical treatments [3]. BT showed similar healing rates at 8 weeks when compared to nitrates [2, 15] but presented the highest recurrence rate of all treatments [2]. The main adverse effect of BT was temporary incontinence, while patients treated with both nitrates and CCB presented headaches that caused treatment cessation in some cases [2]. However, the ideal site, number of injections and dosage of BT have not been established yet [2, 16, 17] and most studies follow-up duration is short [17].

Therefore, the primary objective of the present study was to evaluate the healing rate of AF with incobotulinumtoxinA (incoBoNT/A) at 2 years. Secondary endpoints were healing and improvement rates at earlier time points, anal manometry assessment of IAS, and pain perception, incontinence, and QoL assessment.

## Method

### Study design

Prospective, open-label, single-arm, single-center study carried out in adult patients with AF who were eligible for sphincterotomy surgery. Patients were treated with local incoBoNT/A injection to heal AF with a maximum follow-up of 24 months at the Hospital Nuestra Señora del Prado (Talavera de la Reina, Spain). Eligible patients were older than 18 years with a clinical AF diagnosis for over 2 months that had not responded to previous treatments consisting of dietary and behavioral modifications, analgesics, and local treatment with CCB (diltiazem 2%) or nitrates (trinitrate glyceryl 0.4%). The exclusion criteria were: (1) non-idiopathic AF (also called secondary, present a clear underlying cause), (2) previously untreated patients, (3) BT contraindication such as myasthenia gravis, Eaton-Lambert syndrome, pregnancy, and acetylcholine deficiency. Procedures were approved by the local Ethics Committee and all patients signed an appropriate informed consent, in which they were also informed of the possibility of requiring a re-injection or additional surgery if they do not respond adequately or if they experience a relapse after initially responding well to the treatment.

### Intervention

A vial of 50 U of BT free from complexing proteins (IncoBoNT/A; Xeomin®, Merz Pharmaceuticals GmbH) was diluted in 1.25 ml normal saline, and 3 syringes were filled with 0.4 ml each (16 U). Patients were placed in the lithotomy position and pretreated with topical local anesthesia (prilocaine/lidocaine cream 25 mg/g each). A mean of 40.5 U IncoBoNT/A was injected per patient at 3 sites (spread equally across these three sites): both lateral and posterior intersphincteric groove. After injection, patients were discharged after verifying that there were no complications.

To perform the anal manometry, the THD® Anopress portable system was used. It consists of disposable probes that allow for the simple and quick evaluation of anal pressure values at rest, during voluntary contraction, and during expulsion. This establishes a curve of defecatory dynamics, enabling us to understand these pressures and also to rule out anismus.

### Study endpoints

The primary endpoint was the healing rate at 2 years. Healing was defined as scarred AF and without symptoms; improvement was considered when the fissure persisted without symptoms; recurrence was defined as the return of symptoms similar to those present before the injection, occurring after a cure. Treatment failure refered to patients who required surgical intervention due to the absence of any clinical improvement after treatment.

Secondary endpoints included: healing and improvement rates (based on an examination of the epithelialization of the fissure in the consultation room) at earlier time points, anal manometry assessment of IAS (anal resting pressure), voluntary squeeze pressure and pressure during Valsalva maneuver [VM]), pain perception assessment with a pain visual analog scale (VAS) (scoring between 0 [no pain] and 10 [severe pain]) [18], incontinence assessment with the Wexner score (scoring between 0 [no incontinence] and 20 [total incontinence]) [19], psychiatric condition evaluated by recording any condition or medication through anamnesis and reviewing the patient´s medical history, and QoL assessment with SF-36 questionnaire (scoring between 0 [worst health status] and 100 [best health status]) [18]. Safety was also evaluated throughout the study.

### Timing

Healing/improvement was assessed at 1 week, 1 month, 3 months, 6 months, 1 year, and 2 years post-injection. IAS pressures were measured using THD® Anopress system at baseline and at 1 and 3 months post-injection. Pain perception and incontinence were assessed at baseline, 1 month, 3 months, 6 months, 1 year, and 2 years post-injection. Pain perception was also measured at 1 week, and QoL assessment was carried out at baseline and 3 months post-injection.

### Statistical analysis

Continuous variables are expressed as mean and 95% confidence interval (CI), whereas categorical ones as absolute and relative frequencies. Comparison between baseline and different time points after treatment was performed with Student t-test (in case the data follow a normal distribution) and Wilcoxon Signed-Rank test (if dependent variable is not normally distributed). Statistical significance threshold was *p* < 0.05. All statistical procedures were performed with SAS 9.4 software (SAS Institute, Cary NC, USA).

## Results

### Study population

A total of 49 patients with AF out of 55 initially screened between September 2017 and October 2021 were treated with incoBoNT/A. Causes for exclusion were screening failure (2 patients) and loss to follow-up (4 patients). Treatment with incoBoNT/A was carried out between September 2017 and March 2022. Analysis was based on June 1st 2023 data cut available, with 31 patients having finished the 2-years study and 18 drop-outs, being the median follow-up time 24 months (interquartile range: 12.1–25.9). Figure S1 provides a summary of the study population.

### Baseline data

The mean age of the patients was 47.3 years (range: 26–81) including 61.2% of female subjects. During a mean of 9.6 months (range:1–60), 48 patients (98.0%) had been previously treated with dietary and behavioral modifications, 48 (98.0%) with painkillers, 43 (87.8%) with topical CCB and 35 (71.4%) with topical nitrates. In all cases, those approaches failed to heal the AF. Most AFs were located at the posterior midline (75.5%), and the rest were found in the anterior midline (12.2%) or in both locations (12.2%). Most patients presented bleeding and pruritus (81.6% and 83.7%, respectively). The mean baseline pain VAS score was 7.9 (SD 2.3) and the mean baseline Wexner score was 1.0 (SD 2.2). As for anal manometry data, mean anal resting pressure, voluntary squeeze pressure, and pressure during VM were 53.1 mmHg (range: 22.0–94.0), 108.8 mmHg (range: 38.0–230.0), and 35.1 mmHg (range: 4.0–86.0) at baseline, respectively. Demographic and baseline data are detailed in Table S1. All patients were injected incoBoNT/A at the three respective sites (in posterior intersphyncteric groove and on both laterals). The mean dose administered was 40.5 U (range 32–48).

### Primary endpoint: Healing rate

**Fig. 1:**
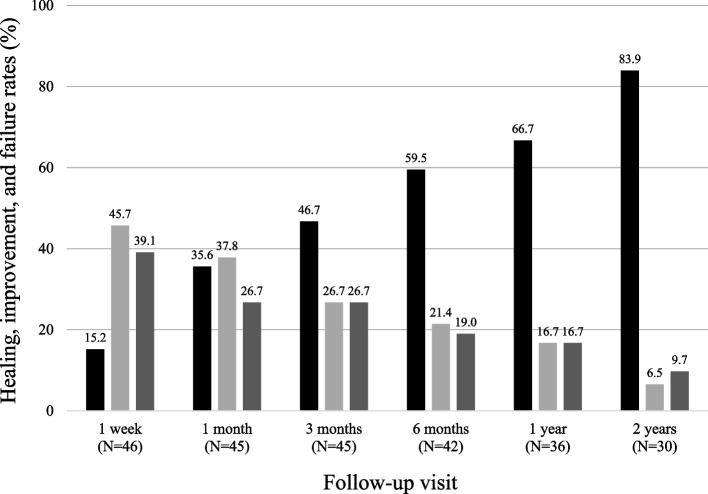
Healing, improvement, and failure rates at different follow-up visits. Healing rates are shown in black, improvement rates are shown in light grey, and failure rates are shown in dark grey. The number of patients (N) that attended each visit is given in brackets

In total, 26 out of 31 patients who attended the 2-year monitoring visit healed (83.9%, 95% CI: 66.3–94.5) (Fig. 1). Taking the entire population into account (N = 49), a total of 32 patients healed at any point during the study (65.3%; 95% CI: 50.3–78.3).

### Secondary endpoints

**Fig. 2:**
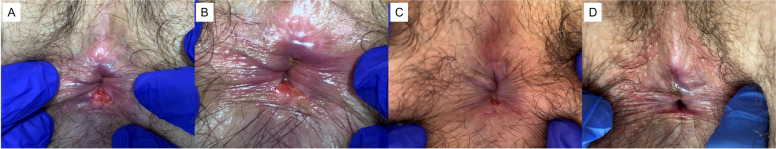
Representative photographs of a patient suffering from chronic AF (**a**) before (baseline), (**b**) one month, (**c**) three months, and (**d**) six months after treatment with incoBoNT/A

During follow-up visits, healing/improvement rate was maintained at over 70%: 73.3% (33/45) subjects improved or healed at 1 month, 73.3% (33/45) at 3 months, 80.9% at 6 months (34/42), 83.3% (30/36) at 1 year, and 90.3% (28/31) at 2 years (Fig. 1). The effective healing after treatment with incoBoNT/A is illustrated in Fig. 2. In total, 20 patients improved or healed throughout follow-up (40.8%), 8 patients (16.3%) had a late healing after 3 months, and in 4 patients (8.2%) incoBoNT/A local injection failed to treat AF during the first week but improved or healed afterwards. There was a total of 12 recurrences (24.5%, 95% CI: 13.3–38.9), with a mean time to recurrence of 9.0 months (range 3–20). Three patients who presented recurrences were re-administered incoBoNT/A and healed, and 9 patients were treated as follows: internal lateral sphincterotomy in two cases, with an inflammatory polyp removal in one of them, fistulectomy with hemorrhoidectomy in a patient with a healed AF who developed a subfisseal fistula and hypertrophied papilla, one patient presented pudendal neuralgia (not secondary to the puncture, but was the real cause of his anal pain) and was treated with specific medication and is improving, another patient showed persistent pain and unspecific inflammatory proctitis found under general anesthesia exploration, and the four remaining subjects presented mild intermittent recurrences but declined surgical treatment. Treatment failed in 50.0% (7/14) and 21.8% of patients with and without psychiatric disorders (anxiety, depression, bipolar disease and adjustment disorders), respectively. Regarding sex, treatment failure was observed in 44.4% (8/18) of men and 21.4% (6/28) of women. Multivariate logistic regression analysis adjusted by age, sex, and psychiatric disorders yielded a probability of failure 8.6 times higher (95% CI: 1.4–52.6; *p* = 0.019) among patients with psychiatric disorders in subjects of the same age and sex. Also, the probability of failure in patients with the same age and psychiatric condition was 6.9 times higher (95% CI: 1.2–38.3; *p* = 0.032) among men.

The mean reduction in anal resting pressure was -7.8 mmHg (SD 14.8; *p* = 0.001) at 1 month and -9.1 mmHg (SD 14.8; *p* = 0.001) at 3 months (Table 1). The mean reduction of voluntary squeeze pressure was -40.9 mmHg (SD 41.6; *p* < 0.001) at 1 month and -27.5 mmHg (SD 34.5; *p* < 0.001) at 3 months. The mean reduction of pressure during VM was -8.6 mmHg (SD 21.3; *p* = 0.012) at 1 month and -13.6 mmHg (SD 17.0; *p* = 0.001) at 3 months. Mean pain perception decreased throughout the study: the mean reduction of pain VAS score was -4.6 (SD 3.1; *p* < 0.001) at 1 week and -6.5 (SD 3.4; *p* < 0.001) at 2 years (Table 2). There was a transient flatus incontinence increase of 1.3 points (SD 3.4; *p* = 0.006) on the Wexner scale at 1 month (mean value 2.3, SD 3.8) when compared to baseline (mean value 1.0, SD 2.2) that was maintained at 3 months (mean value at 3 months 2.0, SD 4.0; 1.0 point difference compared to baseline [SD 3.2; *p* = 0.0586]). Baseline values of incontinence were restored at 6 months (mean value 1.5, SD 3.3) with a trend to decrease at 1 (mean value 0.5, SD 2.3) and 2 years (mean value 0.77, SD 2.2) (*p* = 0.430 and *p* = 0.500, respectively). IncoBoNT/A local injection showed no significant changes in QoL other than an increase in health transition of 23.3 points (SD 32.0; *p* = 0.009) and in bodily pain of 7.9 points (SD 30.6; *p* = 0.044) at 3 months.

**Table 1:** Manometry values

	**N**	**Mean (mmHg)**	**SD**	***p* value**
*Anal resting pressure*				
Baseline	49	53.1	15.5	
1 month	43	45.2	14.6	
3 months	40	45.4	14.1	
Change at 1 month	43	-7.8	14.8	0.001^*^
Change at 3 months	40	-9.1	14.8	0.001^*^
*Voluntary squeeze pressure*				
Baseline	49	108.8	43.7	
1 month	43	65.7	22.9	
3 months	40	82.0	43.2	
Change at 1 month	43	-40.9	41.6	< 0.001^*^
Change at 3 months	40	-27.5	34.5	< 0.001^*^
*Pressure during VM*				
Baseline	49	35.1	19.6	
1 month	43	26.7	16.0	
3 months	40	24.9	14.8	
Change at 1 month	43	-8.6	21.3	0.012^**^
Change at 3 months	40	-13.6	17.0	0.001^**^

*SD* standard deviation, *VM* Valsalva maneuver, *: paired samples t-Student, **: Wilcoxon Signed-Rank Test

**Table 2:** Pain VAS score values

	**N**	**Mean score**	**SD**	*p* value^*^
Baseline	49	7.9	2.3	
1 week	46	3.4	2.6	
1 month	45	2.9	3.1	
3 months	45	3.2	3.3	
6 months	42	2.5	2.8	
1 year	36	2.0	2.8	
2 years	31	1.5	2.2	
Change at 1 week	46	-4.6	3.1	< 0.001
Change at 1 month	45	-5.0	3.4	< 0.001
Change at 3 months	45	-4.6	3.9	< 0.001
Change at 6 months	42	-5.4	3.3	< 0.001
Change at 1 year	36	-5.6	3.0	< 0.001
Change at 2 years	31	-6.5	3.4	< 0.001

*SD* standard deviation, *VAS*: visual analog scale of 0–10, where 0 means no pain and 10 means severe pain, *: Paired-Samples t-Student and Wilcoxon Signed-Rank Test

### Safety

A total of 7 AEs (Clavien-Dindo II) were reported in 7 subjects (14.3%) during follow-up: constipation with consequent increase in pain and posterior mild incontinence at 3 months, hyperemia and inflammatory polyp at 3 months, bruising at 3 months, two patients with subfissure fistula at 3 months, and two patients with thrombosis of external hemorrhoid (at 1 month and 72 h after treatment, respectively). All AEs were treated with ointments and only 3 of them led to treatment failure.

## Discussion and conclusions

Lateral internal sphincterotomy can be safely offered as first-line therapy in selected patients pharmacologically naive with no underlying FI (ASCRS grade 1A) [3]. Although its healing rate ranges from 88 to 100%, anal incontinence after surgery occurs frequently (8–30%) and is permanent in some patients [3, 20]. The risk of permanent incontinence dictates the need for a treatment with no persistent negative effects, and local BT injection seems to be a good candidate since it is a less invasive and safe procedure [16]. The healing rate with BT at 2 months was as low as 29.2% and reached values up to 96% [2, 16, 17, 21]. In a voluntary survey addressed to all ASCRS members, 89.4% of respondents injected 50–100 U of BT to treat AFs [22]. Studies conducted thus far are heterogeneous regarding techniques, BT doses injected, follow-up time, and results [2, 17], leading to a recommendation based on moderate-quality evidence (ASCRS grade 1B) [3]. In fact, in a retrospective study conducted by Brisinda et al. in 1,003 patients with symptomatic chronic AF, the dosage of toxin and the site of injection had an impact on the healing rate [21]. The dose significantly correlated with healing at 2 months, with 29.2%, 75%, 79.3%, and 83.9% healed patients treated with 15 U, 20 U, 30 U, and 50 U of BT, respectively (*p* < 0.001) [21]. Of note, a high-dose circumferential chemodenervation-IAS approach using 100 U of BT yielded a healing rate of 90.7% at 3 months [23]. Moreover, healing had a significant correlation with the site of injection as well (healing rates at 2 months were 59.2% and 82.1% in the posterior and anterior injection, respectively, *p* < 0.001) [21]. Recurrence of healed AF with BT was 55% at 3 years [24] and mean resting anal pressure at 2 months post-BT injection (71.1 ± 16.2 mmHg) was significantly reduced when compared to baseline mean value (96.1 ± 18.0 mmHg; *p* < 0.001) [25]. A higher incidence of treatment failures was observed in men, which may be attributed to the presence of greater sphincter hypertrophy during baseline physical examinations and increased hypertonia in initial manometry. It may be advisable to consider increasing the dosage for these patients. Treatment with BT resulted in a significant improvement in both pain intensity score (variation: -4.2 ± 2.9; *p* < 0.001) and pain post-defecation score (variation: -5.1 ± 3.0; *p* < 0.001) [26]. Another study established an 82% rate of reduction in pain scores defined as minimum reduction in discomfort of 50% in AF patients treated with BT [16]. In our study, the healing rate measured at the 2-year visit was 83.9%. Given the entire population (N = 49), healing rate at the last monitoring visit was 65.3%. Also, improvement/healing rate was maintained over 70% during follow-up visits starting at 1 month. All those values are within the range of healing rates at 2 months obtained in previous studies (range: 29.2%-96%) [2, 16, 17, 21]. Therefore, our results suggest that the efficacy is maintained at 2 years. However, follow-up was difficult due to COVID-19 pandemic and only 31 patients out of the initial 49 attended the 2-year visit. Although some studies suggest that dose and site of injection do not have an influence on BT injection outcome [27, 28], the only differences in treatment between our study and the one by Arroyo et al. [24] were precisely dosage and site of injection. A mean dosage of 40.5 U was used in the present study, administered in both lateral commissures, and in the posterior one, while Arroyo et al. used 25 U injected in both lateral commissures, and in the anterior commissure, yielding a higher recurrence rate at 24 months (55%) [24]. A recent study comparing sites of BT injection found no differences in long-term healing rates while showing differences in postoperative pain scores that yielded better patient satisfaction [29]. In accordance with other studies [24–26], all pressures measured in the manometry (anal resting pressure, voluntary squeeze pressure, and pressure during VM) were significantly reduced compared to baseline both at first- and third-month visits. Our results show a greater reduction in voluntary contraction pressures (related to the external sphincter) at 3 months. Moreover, basal pressure values (related to the internal sphincter and therefore to fissure healing) also improved. This demonstrates that, even if BT is injected at the intersphyncteric level, its paralyzing effect is observed in both sphincters. On this basis, it could be theorized that, if the aim of the treatment is to relax only the internal sphincter, BT could be injected into the muscular mass of the IAS and not at the intersphincteric level. Those results agree with the literature where BT injection induces an improvement in pain under several assessment techniques [16, 30]. Regarding safety, the main AEs previously reported were temporary incontinence, perianal hematoma and thrombosis, thrombosis of external hemorrhoids, prolapse of internal hemorrhoids, and perianal abscess [17]. In the present study, only 7 patients (14.3%) reported AEs, all of which have been previously described. Finally, incontinence was assessed in our study with the Wexner scale score. In accordance with previous studies where incontinence was reported in 5% of patients at 2 months but was not present at 6 months anymore [24], there was a slight increase in incontinence at the first month post-injection that was not further maintained.

Study limitation was the monocentric design which could induce selection bias and limit the external validation so that the results must be interpreted carefully. Furthermore, the heterogeneity and small sample size limit both the theoretical and statistical robustness of the study, particularly with regard to the logistic regressions, as these factors may compromise both the external and internal validity of the findings. Additionally, the effectiveness of the treatment must be interpreted with caution due to the absence of a control group.

In conclusion, this study provides further evidence to support the use of incoBoNT/A injections to treat AFs that persist after treatment with the conservative approach and topical nitrates/CCB. BT injection is a non-invasive treatment that should be considered before other invasive treatments due to its efficacy and safety which includes no permanent impairment. However, on the basis of the mentioned limitations, further research involving more homogeneous populations is necessary to draw more definitive conclusions regarding the treatment's efficacy.

## Supplementary Information

Supplementary Material 1.

Supplementary Material 2.

## Data Availability

The datasets generated during and/or analyzed during the current study are available from the corresponding author on reasonable request.

## Abbreviations

AFs: Anal fissures
IncoBoNT/A: IncobotulinumtoxinA
AEs: Adverse events
IAS: Internal anal sphincter
ASCRS: American Society of Colon and Rectal Surgeons
BT: Botulinum toxin type A
CCB: Calcium channel blockers
VM: Valsalva maneuver
VAS: Visual analog scale
CI: Confidence interval
LIS: Lateral internal sphincterotomy
SD: Standard deviation
