# Supplementary material for: Is chemodenervation with incobotulinumtoxinA an alternative to invasive chronic anal fissure treatments?
Source: BMC Gastroenterol. 2024 Sep 30;24:334. doi: 10.1186/s12876-024-03428-z (PMC11440925; doi:10.1186/s12876-024-03428-z)
Supplement: Supplementary file 1 — Supplementary Material 1. [file 12876_2024_3428_MOESM1_ESM.pptx]

## Slide 1
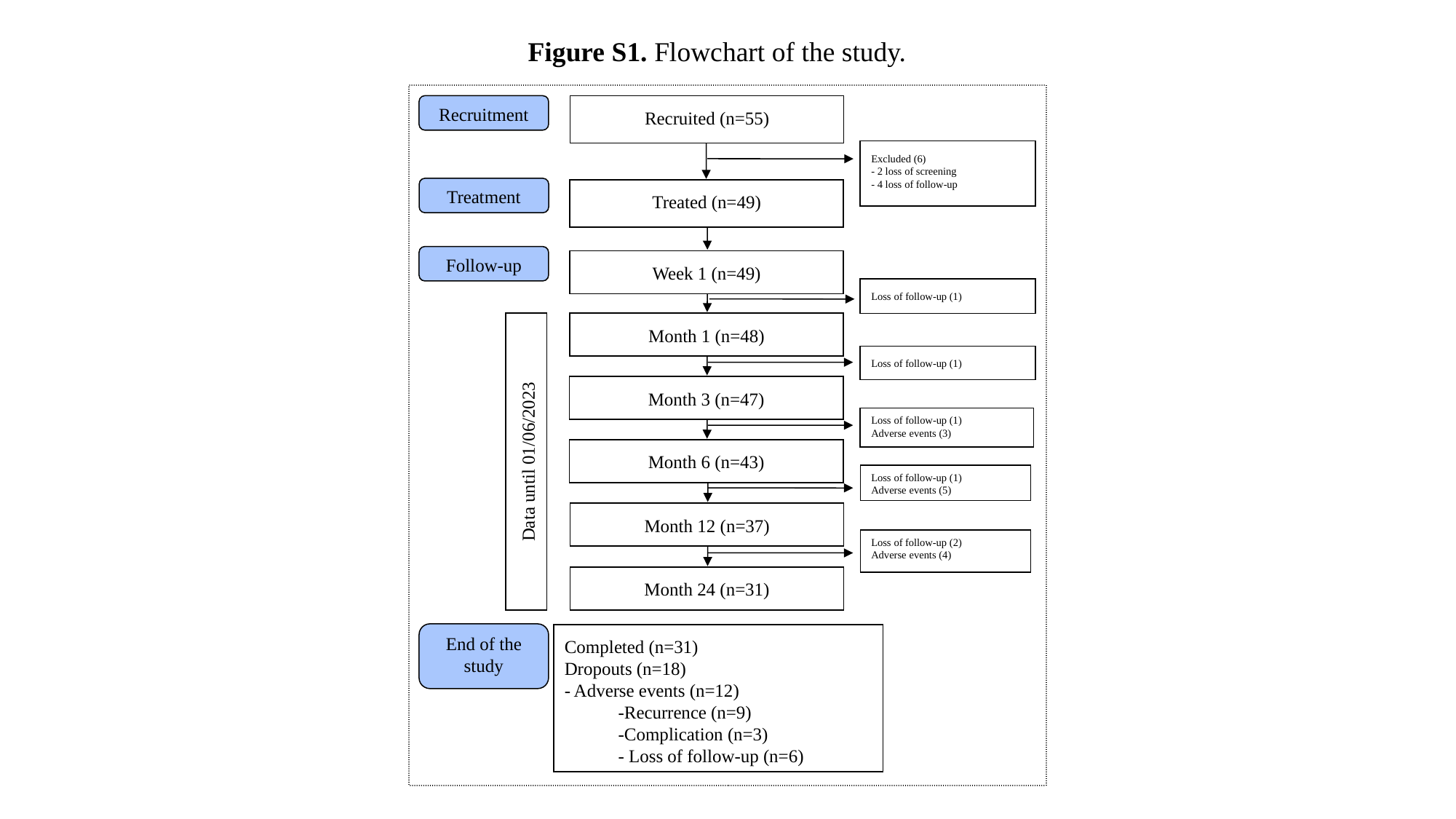

Figure S1. Flowchart of the study.
Recruitment
Recruited (n=55)
Excluded (6)
- 2 loss of screening
- 4 loss of follow-up
Treatment
Treated (n=49)
Follow-up
Week 1 (n=49)
Loss of follow-up (1)
Data until 01/06/2023
Month 1 (n=48)
Loss of follow-up (1)
Month 3 (n=47)
Loss of follow-up (1)
Adverse events (3)
Month 6 (n=43)
Loss of follow-up (1)
Adverse events (5)
Month 12 (n=37)
Loss of follow-up (2)
Adverse events (4)
Month 24 (n=31)
End of the study
Completed (n=31)
Dropouts (n=18)
- Adverse events (n=12)
-Recurrence (n=9)
-Complication (n=3)
- Loss of follow-up (n=6)
