# Supplementary material for: Is chemodenervation with incobotulinumtoxinA an alternative to invasive chronic anal fissure treatments?
Source: BMC Gastroenterol. 2024 Sep 30;24:334. doi: 10.1186/s12876-024-03428-z (PMC11440925; doi:10.1186/s12876-024-03428-z)
Supplement: Supplementary file 2 — Supplementary Material 2. [file 12876_2024_3428_MOESM2_ESM.docx]

**Supplementary material**

**Table S1.** Demographics and baseline data

|  | **Total N=49** |
| --- | --- |
| **Demografic data** |  |
| Age (years), mean (range) | 47.3 (26-81) |
| Males/Females, n (%) | 19 (38.8%) / 30 (61.2%) |
|  |  |
| **Medical history, n (%)** |  |
| Medical background |  |
| Allergies | 10 (20.4%) |
| Antiplatelet/Anticoagulation | 6 (12.2%) |
| Ginecological history | N=30 |
| Deliveries | 19 (63.3%) |
| Instrumental | 5 (26.3%) |
| Episiotomy | 2 (10.5%) |
| Non-instrumental | 12 (63.2%) |
| Cesarean section | 3 (10.0%) |
| Menopause | 11 (36.7%) |
| Urinary incontinence | 1 (3.3%) |
| Urinary history | N=19 |
| Urinary incontinence | 0 (0.0%) |
| Prostatic disease | 3 (15.8%) |
| Anal sexual intercourse | 3 (6.1%) |
| Psychiatric illness | 15 (30.6%) |
| Anxiety-depressive syndrome | 8 (53.3%) |
| Depressive syndrome | 3 (20.0%) |
| Others | 4 (26.7%) |
| Psychiatric illness treatment | 14 (93.3%) |
|  |  |
| **Pathology** |  |
| Fissure location |  |
| Anterior | 6 (12.2%) |
| Posterior | 37 (75.5%) |
| Both | 6 (12.2%) |
| Chronic/recurrent | 49 (100.0%) |
| Duration (months), mean (range) | 16.2 (2-120) |
| Pain (VAS 0-10) , mean (SD) | 7.9 (2.3) |
| Bleeding, n (%) | 40 (81.6%) |
| Itching, n (%) | 41 (83.7%) |
| Wexner scale (0-20), mean (SD) | 1.0 (2.2) |
|  |  |
| **Previous anal fissure treatments, n (%)** |  |
| Radiotherapy in the pelvic area | 2 (4.1%) |
| Anal surgeries |  |
| Fistulas | 0 (0.0%) |
| Anal fissure | 5 (10.2%) |
| Hemorrhoids | 2 (4.1%) |
| Painkillers | 48 (98.0%) |
| Hygienic-dietary measures | 48 (98.0%) |
| Calcium antagonist ointments | 43 (87.8%) |
| Glycerin trinitrate ointments | 35 (71.4%) |
| Others | 9 (18.4%) |
| Treatment duration (months), mean (range) | 9.62 (1-60) |
|  |  |
| **Physical examination, n (%)** |  |
| Hypertrophied papilla | 12 (24.5%) |
| Sentinel hemorrhoid | 10 (20.4%) |
| Sphincter hypertrophy | 33 (67.3%) |
|  |  |
| **Additional examination** |  |
| Endoanal ultrasound, n (%) |  |
| EAS defect | 2 (4.1%) |
| IAS defect | 4 (8.2%) |
| IAS hypertrophy | 10 (20.4%) |
| Anal manometry (mmHg), mean (range) |  |
| Resting | 53.1 (22-94) |
| Voluntary contraction | 108.8 (38-230) |
| Pressure with Valsalva | 35.1 (4-86) |
|  |  |

Abbreviatures: EAS, external anal sphincter; IAS, internal anal sphincter; SD, standard deviation; VAT, Visual Analogue Thermometer pain scale.
